# The Effect of the Feed Direction on the Micro- and Macro Accuracy of 3D Ball-end Milling of Chromium-Molybdenum Alloy Steel

**DOI:** 10.3390/ma12244038

**Published:** 2019-12-04

**Authors:** Balázs Mikó, Bálint Varga, Wojciech Zębala

**Affiliations:** 1 Institute of Material and Manufacturing Science, Óbuda University, Budapest 1034, Hungary; varga.balint@bgk.uni-obuda.hu; 2 Production Engineering Institute of the Mechanical Faculty, Cracow University of Technology, 31-155 Kraków, Poland; zebala@mech.pk.edu.pl

**Keywords:** free form milling, ball-end milling, geometric tolerance, surface roughness

## Abstract

The machining of free form surfaces is one of the most challenging problems in the field of metal cutting technology. The produced part and machining process should satisfy the working, accuracy, and financial requirements. The accuracy can describe dimensional, geometrical, and surface roughness parameters. In the current article, three of them are investigated in the case of the ball-end milling of a convex and concave cylindrical surface form 42CrMo4 steel alloy. The effect of the tool path direction is investigated and the other cutting parameters are constant. The surface roughness and the geometric error are measured by contact methods. Based on the results, the surface roughness, dimensional error, and the geometrical error mean different aspects of the accuracy, but they are not independent from each other. The investigated input parameters have a similar effect on them. The regression analyses result a very good liner regression for geometric errors and shows the importance of surface roughness.

## 1. Introduction

The machining of free form surfaces is a focused problem in the mould and die industry and in power plant turbines. During the manufacturing process planning there are lot of different aspects: process parameters of the cutting technology, tool parameters, and the tool path must be determined. Moreover, the working requirements, the productivity, the required accuracy and the measuring possibilities must be considered.

Products such as moulds and dies often have complex shapes and very accurate dimensions. These elements are generally made of alloys commonly referred to as hard-to-cut materials. Several types of such materials can be distinguished, depending on their features. The most known types are materials with low thermal conductivity, such as super alloys and titanium, a ductile material such as pure nickel, a material with high hardness and brittleness, such as ceramics [1,2,3]. The characteristics of each type of material have a large impact on the choice of machining methods, conditions, equipment and tools. Materials with unique metallurgical properties—such as titanium, tool steels, stainless steels, hardened steels and other super alloys—were developed to meet the demands of extreme applications.

The ball-end milling technology is the most often used in case of piece production, when different tool path strategies can be used. These strategies have effect on the productivity and the accuracy too. The accuracy of the surface has three aspects. The first is the dimensional accuracy: how accurate the size of the feature is; the second is the geometric accuracy: how we match the machined surface to the theoretical; and the third is the surface roughness. 

The geometric tolerances have increasing importance in the industry, and standards describe the interpretations of the different marks, labels and notations [4,5]. Related to the geometric tolerances, many viewpoints can be investigated. Gosavi and Cudney [6] surveys the mathematical methods of evaluation of the form errors, which can modify the results. The point sampling is the other important field of the evaluation of the geometric errors. Zhao et al. [7] presents a B-spline curve based method while Magdziak and Chamdima Ratnayake [8] use a fuzzy inference system in order to determine the number and the distribution of measure points. In case of scan type measuring, the process parameter should be optimized [9] considering the error of the measure and evaluation.

The surface roughness is a micro scale deviation of the surfaces and standards (ISO 4287 [10], ISO 4288 [11]) define several description parameters and the measuring details. From the viewpoint of machining process planning, the estimation of the surface roughness is necessary. There are three different ways. The first is the geometric approach, when the surface roughness is derived from geometric data. The second is the statistical approach, when surface roughness is estimated by heuristic equations based on experiments. The third approach is a combined method, when heuristic equation is created based on geometric model and experimental data. 

Vyboishchik [12] presents a pure geometric approach to describe the surface topography in case of convex and concave surface ball-end milling. Sekulic et al. [13] show a statistical method whereby coefficients of the regression model are determined by genetic and grey wolf optimizer algorithms. Felhő and Kundrák [14] present a combined method in case of face milling by two types of cutting inserts. The natures of the surface and the milling direction have large effect on the surface roughness, as Rybicki [15] presents. The horizontal surface has the worst quality. Bilek et al. [16] show the effect of different edge geometry of the ball-end milling cutter to the surface roughness. The positive geometry and the smaller feed are favourable in case of 1.2379 hardened tool steel. 

The milling strategy has important effect of the accuracy of the machining, beside the tool geometry, the cutting parameters and the nature of the surface. Izol et al. [17] compares the theoretical scallop height (geometric model) and the real surface roughness in case of a convex test surface which was machined by four different milling strategies. The real surface roughness is better than the scallop height. Magalhales and Ferreira [18] investigate three milling strategies from the viewpoint of surface roughness and productivity in case of a convex spherical test part. The radial strategy ensures the best surface quality and the worst machining time. Pena et al. [19] describe the optimization model of milling process, when the milling strategies are considered. The 5 axis milling ensures new possibilities as Sadilek et al. [20] present. Three types of 3D geometry are machined by 3D and 5D milling. They found that the 5D milling provides better shape accuracy and surface roughness. During the planning of the measuring process of the surface roughness, lot of factors have to be considered, as Farkas and Drégelyi-Kiss [21] present through the investigation of uncertainty of surface roughness measuring of turned and milled surfaces.

On the basis of the above, it is therefore concluded that the quality of the machined surface has different aspects and it is defined by many parameters. Based on the literature the surface roughness is in the focus of the researches, but based on the industrial feedbacks the geometric accuracy has the same importance. 

In our current research the geometric, dimensional accuracy, and surface roughness are investigated in the case of the ball-end milling of free form surfaces. The investigation of free form surface machining has many different aspects, but we focus on the process planning and the application of CAM systems. Our aim is to support the work of CAM programmers in the selection of the most appropriate milling strategy. From this point of view the detailed investigation of the chip removal process or the theoretical definition of cutting forces along the edge of ball-end milling cutter have less importance, and there are not in the foreground of our research.

In the current article, the effect of the milling path direction is investigated on the different errors in case of convex and concave cylindrical surface. In the next chapter the test part, the milling circumstances and the applied equipment are introduced. The measured data and the statistical analyses of them are presented, and the discussion closes the paper.

## 2. Materials and Methods 

**Figure 1:**
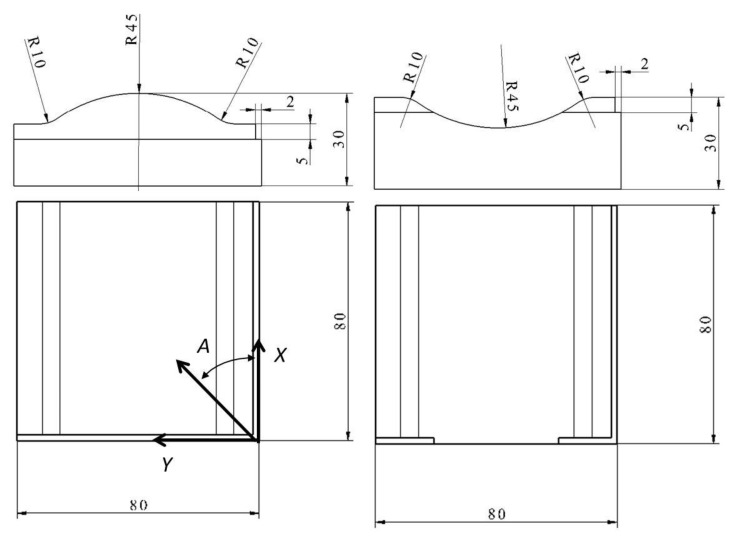
Convex (CX) and concave (CV) test part.

Two types of test part geometries were designed and investigated. The *CX* type specimen contains a convex and the *CV* type specimen contains a concave cylindrical surface. Both radiuses were 45 mm (Figure 1). The normal vector of the surface is changed from 0° to 32.2° related to the vertical direction. The overall dimensions of the test parts were 80 mm × 80 mm × 30 mm. Two shoulders were designed and manufactured to two perpendicular sides of the part, which indicate the x and y directions during measuring process. The two types of test parts (convex and concave) are modelling the different sections of a general free form surface, where convex and concave areas can change each other. The cylindrical surface makes the measuring process easier and comparable.

The material of the test parts was 42CrMo4 (1.7225; Rm = 1000 MPa) low alloy steel (Table 1), which is a chromium, molybdenum, manganese low alloy steels noted for toughness, good torsional strength and good fatigue strength. This grade of steel is often used for statically and dynamically stressed components for vehicles, engines and machines and for parts of larger cross-sections, crankshafts and gears. 42CrMo4 material has good machinability, good wear-resistance, but the temper brittleness is not obvious, and difficult to weld.

The surface was machined by 3D ball-end milling, as a finishing stage, but in order to equal manufacturing allowances a roughing and a pre-finishing were used. During the finishing the applied cutting tool was a Fraisa X7450.450 ball-end milling cutter with 10 mm diameter (D_c_ = 10 mm) and the number of teeth was 4 (z = 4). During the tests the cutting parameters were the same: cutting speed v_c_ = 160 m/min; spindle speed n = 5100 rpm; feed per tooth f_z_ = 0.08 mm; feed speed v_f_ = 1650 mm/min; depth of cut a_p_ = 0.3 mm; width of cut a_e_ = 0.15 mm. The width of cut (a_e_) was same in every set. The width of the cut important effect on the surface roughness, however it was not investigated in this research. The detailed study of this parameter will be the task of the next stage. The width of cut means the distance between the tool paths in *x-y* plane, and it is generated by CAM system. 

The milling of the test surface was done by zig-zag strategy, when the up-milling and down-milling are used alternately in order to higher productivity. The feed direction was changed during the tests. The feed direction was measured to the *x* axis (Figure 1), and five different directions were defined: A = 0°/22.5°/45°/67.5°/90°. The test parts were marked from 1 to 5, so the *CV-4* means the concave test part, where the milling part direction was 67.5° (Table 2). The machining was performed by a Mazak 410 A-II CNC machining centre, the CNC programs were generated by CATIA v5 CAD/CAM system by sweeping method. 

The surface roughness was measured by a Mahr-Perten GD120 contact measurement instrument. The measuring of the surface roughness is complicated in case of free form surfaces, because the measured values depend on the measuring direction. Theoretically the largest value can be measured in the perpendicular direction to the tool path. The measuring of surface roughness by contact method requires a standard length, where the surface section should be close to a horizontal position (or could be rotated in the horizontal position). However, in the case of free form surfaces, sometimes it is not possible. 

**Figure 2:**
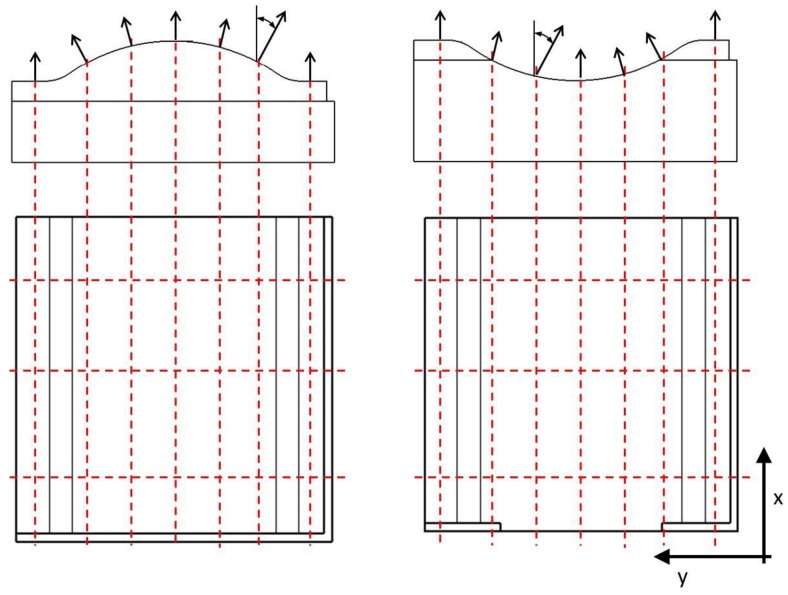
The positions of measuring of surface roughness.

During the investigation the Ra and Rz parameters were evaluated. Measurements were performed in three different directions (AM: angle of the surface roughness measuring). The first was parallel to the *x* axis (AM = 0°), the second was perpendicular to this (AM = 90°), and the third was perpendicular to the feed direction. In some cases, the third case was the same as the first or second. Seven different positions were defined on the surface and they were repeated three times along the length of the part in *x* direction (Figure 2). The angles of the surface normal vector are (in order of marked position) 0°/32.2°/15.5°/0°/−15.5°/−32.2°/0°. In the second and third positions there was not enough space for surface roughness measuring, in those cases only three positions were used. The angles of the surface normal vector are 23.6°/0°/−23.6°. The surface roughness was calculated as average of every measure.

**Figure 3:**
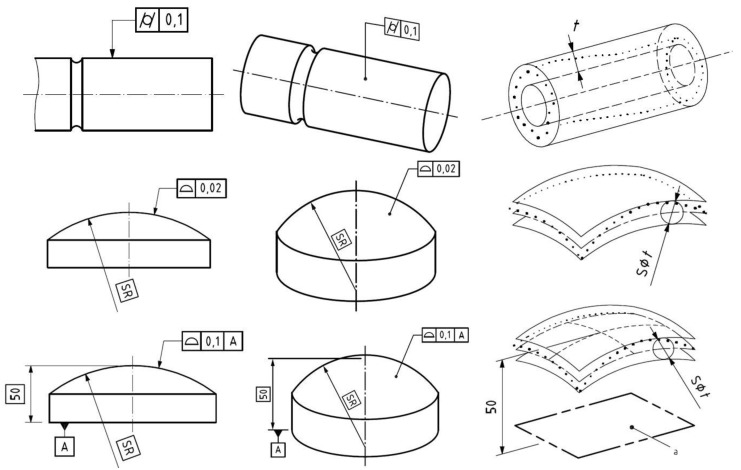
The definition of cylindricity and surface profile error based on ISO 1101 [4].

The focus of the research is on the investigation of geometric tolerances in case of free form machining. Two types of tolerances were selected for investigation considering the properties of the test surface: the cylindricity (Cyl) and the surface profile error (SPE). Based on ISO 1101 standard the cylindricity error is the radial distance of two cylinders (t; Figure 3), which has the same axis and cover the investigated surface. The axis of these cylinders has four degree of freedom, therefore during the evaluation, the algorithm must identify the horizontal and vertical position of the axis and the orientation based on the horizontal and the vertical planes. The cylindricity describes the accuracy of the geometric shape, ignoring its position. The cylindricity tolerance can be applied only in case of cylindrical surfaces. In general case the surface profile error can be used. The surface profile tolerance zone is limited by two surfaces enveloping spheres, the centres of which are situated on a surface having the theoretically exact geometrical form [4]. The defined datum geometries decrease the degree of freedom of the tolerance zone, which results in a stricter requirement and larger calculated error.

The measuring of surfaces’ points was performed by a Mitutoyo Crysta-Plus 544 coordinate measuring machine. A total of 49 points were measured on the surface in 7 × 7 points grids, and in order to help the evaluation datum points were fixed on the sides of the specimens. 

**Figure 4:**
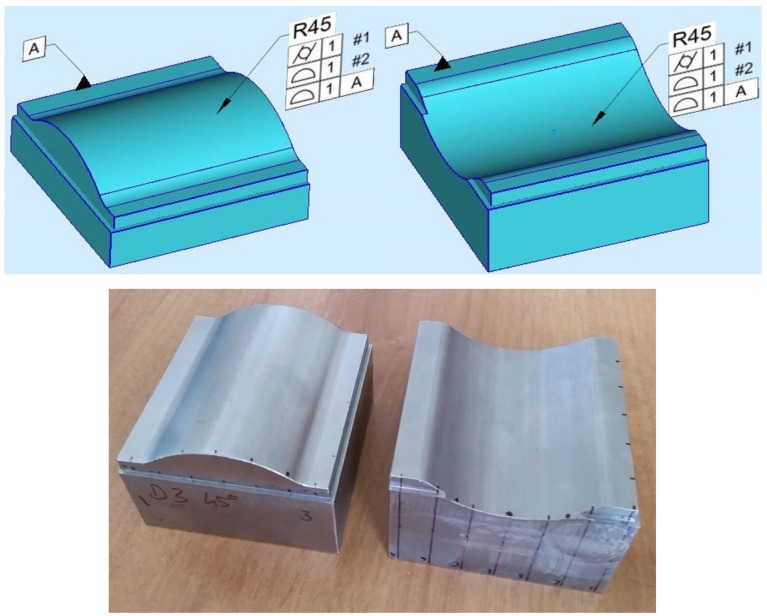
Definitions of geometric tolerances in Smart Profile and test parts (CX-3, CV-3).

The evaluation of the geometric error was determined based on measured points by Kotem Smart Profile v5.0.4.6 software, which can compare the measured point to the theoretical CAD model of the test part. The definitions of geometric tolerances can be seen in Figure 4. The surface profile error was defined without datum and with a plane datum surface. In the first case, the tolerance zone has 6 degrees of freedom (DOF), like in case of cylindricity. Therefore our expectation is that the values of cylindricity and surface profile error will be close to equal. In the second case, the tolerance zone has 3 degrees of freedom, so the value will be larger. During the research a third type of profile error is calculated from the value of the second surface profile error: this is the difference of the minimum and the maximum distance from the theoretical surface. Otherwise the surface profile error is symmetric. The statistical analyses of the results were performed by Minitab v14 software tool.

## 3. Results and Discussion

**Figure 5:**
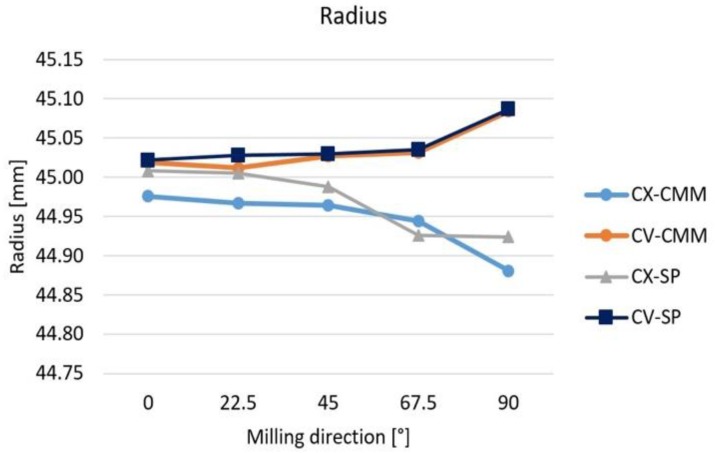
Values of radius of milled surfaces.

The value of the radius was calculated based on measured points by the software of CMM and by the Smart Profile software tool (marked by –CMM and –SP). Figure 5 shows the measured results. The values, which were calculated by different methods, are not differing much from each other. The changing milling path direction has effect on the radius. In the case of the convex part, the radius decreases, whereas in the case of the concave surface the radius increases.

**Figure 6:**
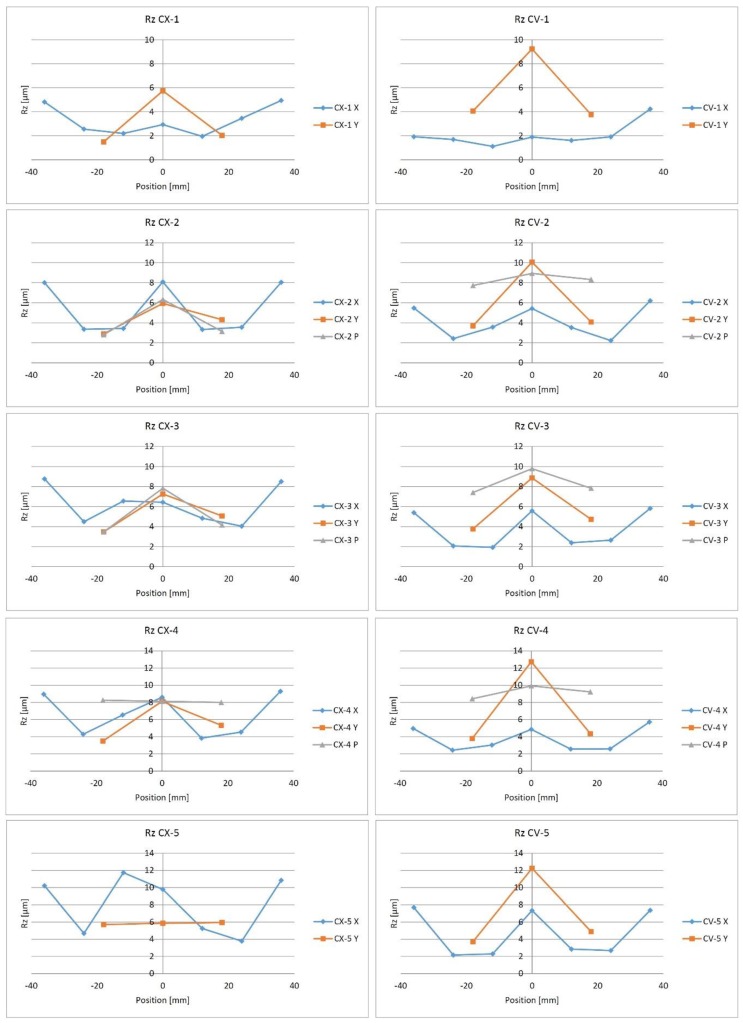
Rz surface roughness in the case of convex and concave test parts in different measuring directions and positions.

In accordance with our previous experiences, the surface roughness is influenced by the surface normal vector: in the case of a horizontal surface the surface roughness is worse. So in case of test parts a typical W form can be observed (Figure 6). *X* and *Y* in the notation of the curves mean the direction of the measure (X – AM = 0°; Y – AM = 90°), and *P* indicates the perpendicular measuring direction to the milling path. The measuring direction modifies the values, because of the nature of the contact measuring method. Theoretically, the largest values will be at the direction perpendicular to the milling.

**Figure 7:**
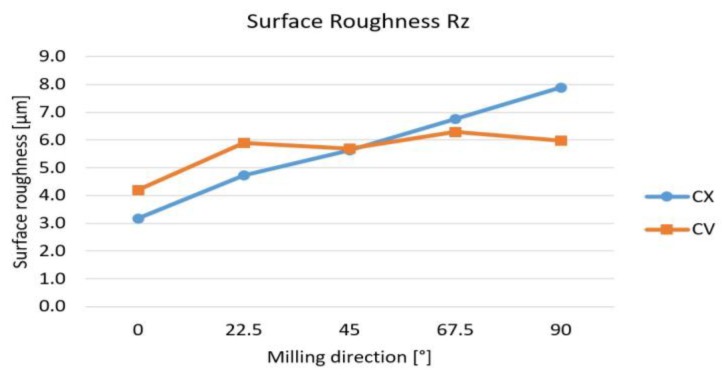
Average values of Rz in function of milling direction.

Based on every measured value of surface roughness the average values can be calculated. Figure 7 shows these values in the function of the milling direction. From *x* milling direction (AM = 0°) to *y* direction (AM = 90°) the surface roughness increases, but in the case of a convex shape, this process is faster. The average values of the concave surfaces are near constant.

**Figure 8:**
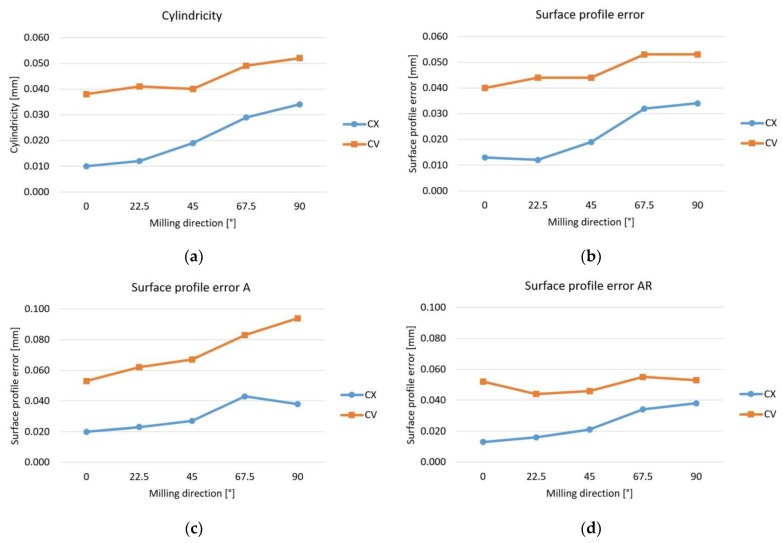
Values of geometric errors. (**a**) Cylindricity error; (**b**) Surface profile error; (**c**) Surface profile error in the case of *A* datum; (**d**) The range of the surface profile error in the case of *A* datum.

The values of the different geometric errors can be seen in Figure 8. In general, the values increase with the changing of feed direction, the smallest values can be observed in case of milling parallel to the *x* axis (AM = 0°), and the largest values are observed when milling parallel to the *y* axis (AM = 90°). The convex surfaces have a better accuracy than the concave.

The values of the cylindricity and surface profile error without a datum surface are very similar, because of the similarity of their definitions. Both have 6 degrees of freedom, but the surface profile error must be symmetric to the theoretical surface, while the cylindricity shows just the width of error zone.

If datum plane is defined (Figure 8c), the DOF decreases and the values are larger, so not just the form deviation is considered, but the position error too (“*A*” means the use of datum plane *A* during the assessment, see Figure 4). Figure 8d shows the width of the error zone in the case of use of the datum plane (*AR* means the use of the datum plane and the range of the error zone is taken into account). The large difference from the diagram of Figure 8c indicates the position error, which is smaller in the case of convex surfaces.

**Figure 9:**
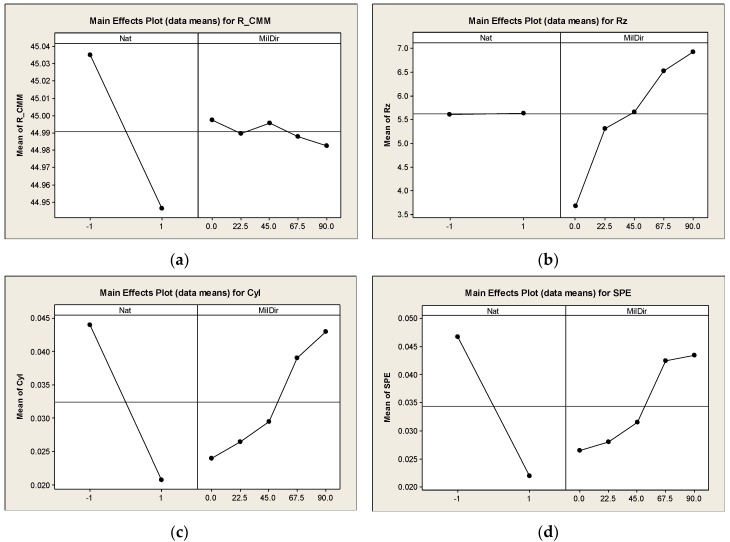
Main effects plots of the measured parameters. (**a**) Main effects plot for the radius; (**b**) Main effects plot for the Rz surface roughness; (**c**) Main effects plot for cylindricity; (**d**) Main effects plot for surface profile error.

The ANOVA analyses of the data are shown in Figure 9. The main effects plots show the average value of the measured parameter in the function of the selected parameter, and they can indicate the importance of it. Based on them, the nature of the surface (Nat: +1/−1 = convex (CX)/concave (CV) surface) has important effect on the radius (R_CMM), cylindricity (Cyl) and the surface profile error (SPE), but has no effect on the surface roughness (Rz). 

The milling path direction (MilDir = A) has an effect on all four parameters. In case of radius (measured by CMM), the values decrease with the changing of milling direction, but comparing with Figure 5, it comes from source of average value. The decreasing of the radius of the convex surface is larger than the increasing of the radius of the concave surface. The milling path direction has larger effect on the surface roughness (Rz), the cylindricity (Cyl), and the surface profile error (SPE).

**Figure 10:**
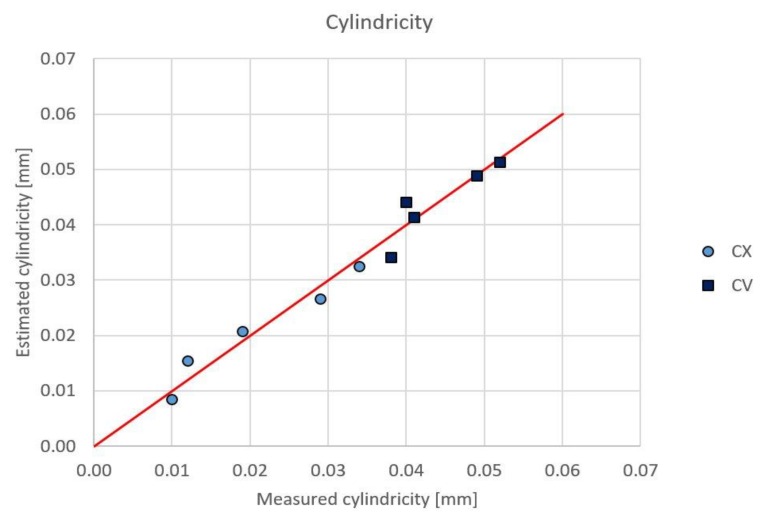
The measured and the estimated values of the cylindricity.

Based on these data regression analyses were performed. The nature of the surface (Nat), the milling path direction (MilDir) and the surface roughness (Rz) were considered for the linear regression generation. In the case of cylindricity (Cyl) the value of R^2^adj is 95.6%, so Equation (1) can describe the cylindricity with good accuracy, as shown in Figure 10. The measured and estimated values are close to the ideal state (continuous red line).

Cyl = 0.0128 – 0.0116 Nat + 0.000145 MilDir + 0.00232 Rz(1)

PredictorCoefSE CoefTPConstant0.012830000.006465001.980.094Nat−0.011632500.00097420−11.940.000MilDir0.000144850.000060732.390.054Rz0.002323000.001531001.520.180S = 0.00307985R-Sq = 97.1%R-Sq = 95.6%

**Figure 11:**
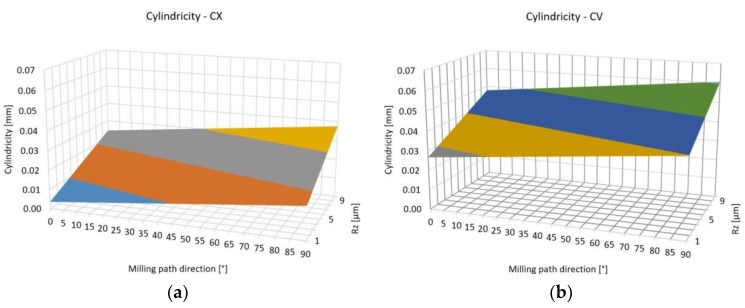
(**a**) Estimated cylindricity in the case of convex surfaces; (**b**) Estimated cylindricity in the case of concave surfaces.

As Figure 11 shows that the convex surfaces have smaller cylindricity error than the concave surfaces. The geometric error and the surface roughness have a close relationship. The milling path direction has an important effect on the geometric error, but the effect of the parameters of the machining process (like the tool diameter, width of cut, the feed speed and the cutting speed) have to be considered. Therefore, the accurate estimation of the surface roughness of free form milling is an important research topic.

The three types of surface profile error can be estimated by similar equations with good accuracy (Table 3).

## 4. Conclusions

The quality of a machine part can be characterized by several properties and parameters, and the tolerance system has design, manufacturing, and measuring aspects which need close cooperation and harmonization. In this research the application of the CAM systems is investigated, and the effect of the tool path parameters are studied in case of free form surface milling by ball-end milling cutter. In the literature the investigation of the geometric tolerances in case of cutting technologies is underrepresented, however it has a large importance in the industry. In the current article, the dimensional accuracy, surface roughness, and geometric error were investigated. Convex and concave surfaces with 45 mm radius were machined by milling in various tool path directions, and the effect of it was investigated on the surface roughness, the dimension error, the cylindricity and the surface profile error.

Based on the results above, it was found that the surface roughness, the dimensional error and the geometrical error mean different aspect of the accuracy, but they are not independent from each other. The investigated input parameters have similar effect on them. 

The range of the measured values of the surface roughness is same like in [13] and [18] in the case of similar circumstances. The effect of the tool path on the surface roughness is presented in [18]. The tool path direction from *x* to *y* axis increased the surface roughness in our research, but the nature of the surfaces (convex or concave) has no effect on it. The cylindricity and the surface profile error were sensitive to the nature of the surface and the milling direction had effect on them. The convex surfaces have smaller geometric errors, due to the working length of cutting edges probably. In the case of concave surfaces, the length of the cutting edge is larger, so the cutting force is larger too, which causes larger geometric errors. The errors were larger in the case of the milling path parallel to the *y* axis.

The dimensional error changed parallel to the milling path direction, but the nature of the surfaces has essential effect on it. Two reasons can be supposed that causes this problem: the inaccuracy of the tool radius, and the changing load of the tool. 

In the case of convex surfaces, the radius of the test parts was decreased; in the case of concave surfaces the radius was increased. The same direction of the tool deformation caused the smaller and the larger surface radiuses on the convex and concave surfaces. In case of milling parallel to the *x* axis, the difference of the measured radius was small, but the difference was larger in case of milling parallel to the *y* axis, therefore the larger cutting force and deformation of the tool were supposed in the case of *y* direction. In the case of the milling parallel to the *x* axis, during one run the working diameter, load of the tool, and tool deformation are constant. But in case of other direction, they are changed in every position of the tool, as [18] presented too. In the case of milling parallel to the *y* axis there is the fastest changing of these parameters. The changing chip cross section and the changing contact cutting edge cause different force and torque on the cutting tool, and the load of the tool causes different tool deformation. The different length of the cutting edge is described in [10] in the case of convex and concave cylindrical surfaces. During the following part of the research the measurement of the cutting forces is required in order to clarify the differences in the tool load and the possible deformation. The difference between the real and the theoretical radius of the cutting tool may be the other source of the dimensional error. If the tool radius is larger than the nominal value, the generated CNC code will result in a smaller convex and larger concave surface. 

The regression analyses gave a good liner regression for geometric errors, where the nature of the surface (+1/−1), the direction of the milling path and the surface roughness were considered. Based on the results, the surface roughness is an important factor, so the estimation of the surface roughness of the ball-end milling is essential. The results of the current stage of the research show that during the selection of an appropriate CAM strategy and the definition of tool path parameters, the nature of the surface should be considered in order to produce accurate free form surfaces. In future work, the effect of the width of cut (step over) parameter and different type of milling strategies will be investigated, and the changing of the cutting force will be considered.

## Figures and Tables

**Table 1:** Chemical composition—42CrMo4 (%).

	C	Si	Mn	Mo	S	Cr	Ni	Other
Result	0.43	0.26	0.65	0.16	0.021	1.07	0.19	Remainder

**Table 2:** Marking of the test parts.

	A				
	0°	22.5°	45°	67.5°	90°
Convex part	CX-1	CX-2	CX-3	CX-4	CX-5
Concave part	CV-1	CV-2	CV-3	CV-4	CV-5

**Table 3:** Regression equations of the surface profile errors.

Equation	R^2^adj
SPE = 0.0147 – 0.0124 Nat + 0.000132 MilDir + 0.00245 Rz	94.4%
SPE_A = 0.0551 – 0.0207 Nat + 0.000521 MilDir – 0.00490 Rz	96.9%
SPE_AR = 0.0146 – 0.0128 Nat + 0.000058 MilDir + 0.00357 Rz	86.5%
